# The Impact of Modern Dietary Practices on Cancer Risk and Progression: A Systematic Review

**DOI:** 10.7759/cureus.46639

**Published:** 2023-10-07

**Authors:** Stephanie Nagy, Stephanie N Petrosky, Michelle Demory Beckler, Marc M Kesselman

**Affiliations:** 1 Rheumatology, Nova Southeastern University Dr. Kiran C. Patel College of Osteopathic Medicine, Davie, USA; 2 Nutrition, Nova Southeastern University Dr. Kiran C. Patel College of Osteopathic Medicine, Davie, USA; 3 Microbiology and Immunology, Nova Southeastern University Dr. Kiran C. Patel College of Allopathic Medicine, Davie, USA

**Keywords:** plant-based diet, ketogenic diet, mediterranean diet, nutrition, diet, cancer

## Abstract

Cancer is a leading cause of mortality around the world, despite continued advancements in the management of cancer. Recent research efforts have shifted to evaluating the role that modifiable risk factors play in cancer risk and development, as diet and nutrition have been found to play a significant role in the onset and progression of cancer. As a result, there has been an increasing focus on the impact of dietary modifications on preventing the onset, progression, and reoccurrence of cancer. In this systematic review, data were collected on three common diets, the Mediterranean diet (MD), ketogenic diet (KD), and plant-based diet, to gain insight into the application of these three dietary modification approaches for risk prevention and limitation of cancer burden. Initially, 4,397 articles were identified from three databases (Ovid, Web of Science, and CINHAL). After removing studies based on the exclusion criteria, only 23 studies were eligible to be included in the systematic review of which 15 evaluated the MD, four assessed the ketogenic diet, and four evaluated the plant-based diet. Each article was considered for its methods, procedures, and findings.

The findings indicate that dietary interventions may effectively reduce the odds of cancer development and the advancement of diagnosed cancers. With the introduction of the MD, KD, and plant-based diets, significant improvements in lowering cancer development, recurrence-free status, and limiting tumor growth were noted across numerous cancer types. Currently, the MD has been extensively studied in the literature, and amongst the widest variety of cancer types. Additional information and evaluation are required on the ketogenic and plant-based diets to fully understand their impact on the cancer burden across a wider subset of cancers. Clinicians should evaluate and recommend nutritional adaptations to their patients to limit the development of specific cancers and as an adjunctive therapy to traditional pharmacological treatment options for patients with diagnosed cancers.

## Introduction and background

In 2020, more than 19 million individuals were diagnosed with cancer and there were 10 million cancer-associated deaths, according to the Global Cancer Statistics [1]. With millions of individuals experiencing the impact of cancer, the current leading cause of mortality worldwide, there has been an increased focus on the modifiable risk factors of obesity, smoking, alcohol, diabetes, sedentary lifestyle, medication use, sun protection, safe sex practices, and diet, which have been shown to play a significant role in the progression and development of cancer [2]. The American Cancer Society (ACS) outlines guidelines for diet and physical exercise to limit cancer progression that includes eating foods high in nutrients, vegetables such as dark green, red, and orange vegetables with fiber-rich legumes, fruits in a variety of colors, and whole grains. The ACS recommends limiting red meats, sugar-sweetened beverages, highly processed foods, and refined carbohydrates [3,4]. Select modern-day diet trends are more commonly used by cancer patients and survivors over strict dietary guideline recommendations [5]. The use of dietary intervention to target the underlying mechanisms of cancer alongside traditional pharmaceutical oncological treatments could be of value to patients.

In the last decade, there has been increasing interest in examining the impact that the Mediterranean diet (MD), ketogenic diet (KD), and plant-based diets may have on the risk and progression of cancer. The MD consists of omega-3-fatty acids, monounsaturated and polyunsaturated fatty acids, green leafy vegetables, fruits, whole grains, legumes, nuts, fish, and poultry, with a limited intake of dairy and processed red meats [6]. The KD consists of high fats, moderate protein, and low carbohydrates, intending to increase the ketone concentration in the body to be used as an alternative energy source for organs. This is because ketones have been shown to act as an antioxidant to reduce free radical damage [7]. Plant-based diets focus on consuming a diet rich in fruits, vegetables, whole grains, nuts, legumes, and seeds and exclude all animal product forms [8].

The goal of this study was to analyze and discuss the clinical impacts of three common modern-day diets, the MD, KD, and plant-based diet, and to summarize the current understanding surrounding the impact diets may have as an adjunctive therapy alongside cancer treatment for patients with cancer.

## Review

Methods

Search Strategy

A systematic literature review was performed using CINHAL, Ovid, and Web of Science for each of the three diets, MD, KD, and plant-based diets. The search was conducted using the Boolean operators “AND” and “OR” between the keywords selected for the literature search of each diet as follows: “Mediterranean diet AND cancer,” “ketogenic diet OR keto diet AND cancer,” and “plant-based diet AND cancer.” The articles were filtered to be within the last decade, 2013-2023, in the English language, and relevance was evaluated in a hierarchical approach that evaluated the title, abstract, and then the full manuscript. For articles that were not freely accessible, the Nova Southeastern University library database was used to gain access.

Selection Criteria

**Figure 1:**
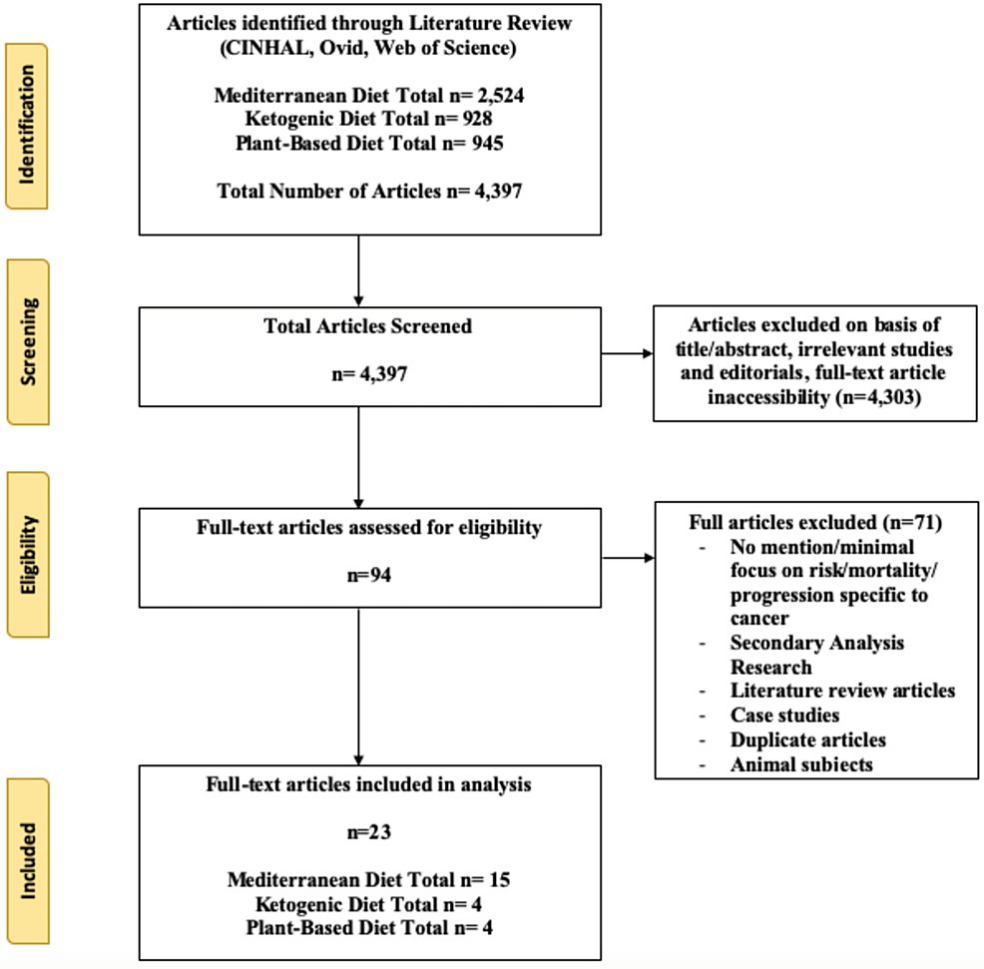
PRISMA chart PRISMA: Preferred Reporting Items for Systematic Reviews and Meta-Analysis

The studies included were considered eligible if they investigated the impact diet had on the risk of cancer development or its effects on cancer progression. Eligible study designs included randomized control trials, case-control studies, and prospective/retrospective studies. Inclusion criteria included free full-text versions, articles in the English language, and the inclusion of keywords within the title and abstract. Exclusion criteria included study designs which were secondary analysis-based studies, case studies and literature reviews, non-in vivo human studies, inaccessibility of the full-text version, duplicate studies, and if the study had no mention of risk/mortality or progression specific to cancer. Each article was screened by a researcher, with a second researcher as a verifier, and a third was assigned to mitigate any disagreements. A flow diagram of the selection criteria (Figure 1) was developed using the updated requirements outlined by the Preferred Reporting Items for Systematic Reviews and Meta-Analysis (PRISMA) [9].

Results

A total of 4,397 articles were identified based on the keyword searches of the three databases (CINHAL, Ovid, and Web of Science). After screening and determining the eligibility of the articles, 23 articles were included. In addition, 4,303 articles were excluded based on their title/abstract, inaccessibility of the full-text version, and if the studies were irrelevant to the established keywords or editorials. Out of the 94 remaining, 71 were excluded based on the exclusion criteria: Study design (secondary analysis-based studies, case studies, or literature reviews), non-human participants, inaccessible full-text articles, duplicate studies, and the lack of mention pertaining to impact on cancer risk or progression. Out of the 23 articles, 15 analyzed the relationship between the MD and cancer, four analyzed the relationship between the KD and cancer, and another four analyzed the relationship between the plant-based diet and cancer. In the literature search for ketogenic and plant-based diets, a significant proportion of results found using the search strategy mentioned above were unrelated to the established keywords; as a result, only a small portion of the articles found were suitable to be assessed further for eligibility and inclusion into the study. Table 1 depicts the studies included in the analysis between the MD and cancer, publication year, subjects, intervention, and significant findings. Table 2 depicts the studies included in the analysis between the KD and cancer, publication year, subjects, intervention, and significant findings. Table 3 depicts the studies included in the analysis between the plant-based diet and cancer, publication year, subjects, intervention, and significant findings.

**Table 1:** Analysis of literature investigating the Mediterranean diet (MD)

Study	Publication Year	Subjects	Intervention	Significant improvements
Mantzorou et al. [10]	2022	114	MedDietScore was used to assess the diets of participants.	MD was found to cause a lower body mass index, earlier disease stages, smaller tumor size, absence of lymph node metastases, longer recurrence-free survival (p ≤ 0.05)
Gardeazabal et al. [11]	2020	10,713	MD pattern diet vs. Western pattern diet evaluated with a 136 item semi-quantitative food frequency questionnaire.	High adherence to the Western pattern diet was associated with an increased risk overall of being diagnosed with breast cancer (p ≤ 0.045). Following a Mediterranean pattern diet was inversely associated with breast cancer development (p≤ 0.045).
Mourouti et al. [12]	2014	250	MedDietScore was used to assess the diets of participants.	Adherence to an MD was inversely associated with the likelihood of having breast cancer in postmenopausal women. Observed that a 1 unit increase in the MedDietScore was associated with 96% lower likelihood of developing breast cancer The most protective factor in the MD against breast cancer was found to be non-refined cereals and red meats were the most harmful factors
Castello et al. [13]	2014	1017	Mediterranean pattern diet vs. Western pattern diet Diets were evaluated using the Alternative Healthy Eating Index and the Alternative Mediterranean Diet Score.	Adherence to MD was related to a lower risk of developing breast cancer, and the Western pattern diet was related to a higher risk of breast cancer. MD had a strong protective effect for triple-negative tumors (p=0.04).
Ricceri et al. [14]	2017	297 women	MD indices were used to evaluate the quality of the MD.	High levels of vegetable intake had significantly lower endometrial cancer risk (p<0.0003). Women who had high levels of adherence to MD has a risk of endometrial cancer half to those that had a low adherence to MD.
Erdrich et al. [15]	2015	12	MD was evaluated using a 14-point Mediterranean diet adherence questionnaire.	Overall adherence to the MD reduced cancer-cell DNA damage significantly (p=0.013).
Castello et al. [16]	2018	1277	Evaluated using a 154-item semiquantitative food frequency questionnaire.	High adherence to MD high in fruit, vegetables, legumes, fish, and olive oil significantly reduced prostate cancer risk (p=0.023)
Stojanovic et al. [17]	2017	223	Assessed using a food frequency questionnaire	Higher adherence to MD resulted in lower gastric cancer risk.
Alvarez-Alvarez et al. [18]	2021	3394	Diet evaluated using the MedDietScore, relative Mediterranean Diet, alternative Mediterranean Diet, Dietary Score and the MEDI-LITE assessment.	Statistically significant decline in gastric cancer risk (p<0.001).
Amiry et al. [19]	2022	270	Willett-format food frequency questionnaire was used to evaluate diet.	Patient consuming a MD had a statistically significant decrease of 52% in the risk of developing gastric cancer (p=0.05). Patient who consumed the MD in the highest quartile had an 83% reduction in gastric cancer than those in the lowest quartile.
Tayyem et al. [20]	2022	486	Arabic food frequency questionnaire used to evaluate diet.	MD diet when consumed in the third and fourth quartile, significantly reduced gastric cancer.
Grosso et al. [21]	2014	338	MedDietScore was used to assess the diets of participants.	Found a significant reduction in the odds of having cancer when adhering to an MD.
Bravi et al. [22]	2018	690	MedDietScore was used to assess the diets of participants.	High adherence to the MD reduced breast cancer risk by 35% compared to low adherence to the MD. High dietary adherence would have led to a 12.6% reduction in overall cancer occurrence (p<0.02).
Gnagnarella et al. [23]	2013	4336	Diet evaluated using Alternative Mediterranean Diet score.	Adherence to a MD was significantly associated with reduced lung cancer risk (p<0.0.45). Significant reduction of lung cancer risk with high consumption of vegetables (p<0.03), fruits (p<0.03), olive oil (p<0.01), fish (p<0.04). Significant increase in lung cancer risk with a high consumption of red meat (p<0.003).
Barrea et al. [24]	2022	391	Diet assessed with the MD scoring system.	High risk of developing thyroid malignancies was significantly associated with a low adherence to MD (p<0.001). Development of thyroid nodules was significantly associated with low adherence to MC (p<0.001).

**Table 2:** Analysis of literature investigating the ketogenic diet (KD)

Study	Publication Year	Subjects	Intervention	Significant improvements
Khodabakshi et al. [25]	2021	80	KD	KD led to a significant decrease in tumor size, and stage of breast cancer (p=0.01)
Khodabakshi et al. [26]	2020	60	KD	A higher survival rate in patients consuming a KD (p=0.04)
Toorang et al. [27]	2021	178	KD Calculated score using a Diet History Questionnaire	Found no statistically significance between a KD and reducing the risks of gastric cancer (p<0.26).
Furukawa et al. [28]	2019	10	Chemotherapy-ketogenic diet vs. chemotherapy alone	Chemotherapy-ketogenic diet group experienced higher disease control rates, better response to therapy and longer overall survival rates.

**Table 3:** Analysis of literature investigating the plant-based diet

Study	Publication Year	Subjects	Intervention	Significant improvements
Sasanfar et al. [29]	2021	421	Plant-based diet Healthy and unhealthy plant-based diet indexes were examined with 168-item food frequency questionnaire	Eating a healthful plant-based diet had an inverse effect on the risk of breast cancer (p=0.002)
Rigi et al. [30]	2021	350	Plant-based diet Healthy and unhealthy plant-based diet indexes were examined with a questionnaire.	High adherence to plant-based diets reduced the odds of having breast cancer by 77%. Those who consume an unhealthy plant-based diet were 2.12 times more likely to develop breast cancer.
Ratjen et al. [31]	2021	1404	Plant-based diet Diet analyzed via a 112-item food frequency questionnaire.	Consuming a plant-based diet significantly reduced mortality rates.
Liu et al. [32]	2021	156	Plant-based diets vs. animal-based diets Dietary intake was assessed every four years using food frequency questionnaires.	Plant-based low-carbohydrate diet scores were inversely associated with hepatocellular carcinoma risk (p=0.03)

MD impact on cancer progression and risk

Breast Cancer

Mantzorou et al. evaluated the impact of adherence to the MD on the progression and recurrence of breast cancer. The study included 114 women aged 35-87, 80 percent with ductal breast carcinoma and 20 percent with lobular breast carcinoma. Patients completed the Mini Nutrition Assessment questionnaire regarding their nutrition status. Their adherence to the MD was evaluated with the MedDietScore, physical activity level, and body mass index. Participants were followed for 42 months or until the breast cancer reoccurred. After the follow-up marker, 22 patients had relapsed at an average of 38 months. The higher adherence to the MD was found to have a smaller tumor size (p<0.017), absence of nodular metastases (p<0.026), recurrence-free survival (p<0.001), and earlier disease states (p<0.008). In addition, they were seen to have lower body mass index (BMI; p<0.001), significantly better physical activity levels (p<0.015), and nutritional status (p<0.036). Breast cancer survivors with higher adherence to the diet and well-nourished patients were found to be independently related to longer recurrence-free survival (p<0.05). This study highlights the need to focus investigations on the impact of adherence to the MD before and after therapy on cancer reoccurrence and progression, in addition to evaluating adequate nutrition status [10].

Gardeazabel et al. analyzed the breast cancer risk in a large-scale prospective cohort study of 10,713 young and middle-aged women followed for 10.3 years. In the follow-up period, 100 confirmed and 168 probable breast cancer cases were identified. Patients were separated based on their adherence to Western or Mediterranean dietary patterns determined by a 136-item semi-quantitative food frequency questionnaire. The Western-based diet was classified as higher in whole-fat dairy, processed meals, fast foods, processed and unprocessed red meats, potatoes, and low consumption of fruits, vegetables, whole grains, low-fat dairy products, and fish. The MD was classified as having high consumption of vegetables, fruits, legumes, nuts, eggs, fish, olive oil, and potatoes. Higher adherence to the Western-based diet was associated with a significantly increased risk for breast cancer (p<0.045), and the MD was inversely associated with breast cancer occurrence in pre-menopausal women. Conversely, a higher adherence to a Western-based diet increased the overall risk of breast cancer development (p<0.045). The study identifies a recommended shift to the MD over the Western diet, which could reduce the risks of breast cancer development [11]. 

Mourouti et al. completed a case-control study of 250 newly diagnosed breast cancer female patients and one-to-one matched controls. Patients were evaluated on their sociodemographic, clinical, lifestyle, and dietary characteristics using a questionnaire. Their adherence to the MD was assessed using the MedDietScore system. A one-unit increase in the MedDietScore, reflecting greater adherence to the MD, resulted in a 96% lower likelihood of developing breast cancer (p<0.05). Non-refined cereals, vegetables, fruits, and alcohol result in the greatest protection against breast cancer; however, alcohol and red meats increased breast cancer development. Overall, adherence to the MD was inversely associated with the development of breast cancer in postmenopausal women [12]. 

Castello et al. conducted a case-control study matching 1,017 patients with breast cancer as compared with controls. Breast-cancer patients were divided based on their breast cancer pathology into either epidermal growth factor receptor 2 (HER2)-negative tumors, HER2+ tumors, or triple-negative tumor types. The dietary intake of the patients was evaluated for the previous five years using a 117-item semiquantitative food frequency questionnaire. The Alternative Healthy Eating Index and the Alternative Mediterranean Diet Score were used to assess the quality of the patient’s diet. Higher adherence to Western dietary patterns resulted in a higher risk of breast cancer, most significantly in premenopausal women. The MD was associated with a lower breast cancer risk and had a stronger protective factor in reducing the size of tumors with triple-negative somatic mutations (p<0.04). Overall, the investigators concluded that shifting to an MD diet could act as a protective factor against the progression of tumors and the development of breast cancer [13]. 

Endometrial Cancer

Ricceri et al. analyzed 297 women with endometrial cancer and matched them with 307 controls in a case-control design. Consumption of fruits and vegetables, adherence to the MD, and the dietary inflammatory index were investigated for their effect on endometrial cancer risk, applying logistic regression for data analysis of the results. The dietary inflammatory index classified eight dietary habits, including a high monounsaturated/saturated fat consumption ratio, high consumption of legumes, cereals, fruits, and vegetables, moderate alcohol intake, and low consumption of meat and dairy products. Low adherence to the MD was classified as zero to three habits, moderate was four to five habits, and high adherence was more than six habits. The researchers found that a higher quantity of vegetable intake significantly lowered the odds of developing endometrial cancer (p<0.0003), and women who had a high to moderate adherence to the MD had half the likelihood of developing endometrial cancer compared to those with a poor adherence. The investigators identified potential protective factors that following an MD, especially one high in vegetables, can have in reducing endometrial cancer risk [14]. 

Prostate Cancer

Erdich et al. evaluated the adherence to the MD in 20 men diagnosed with prostate cancer over three months. The men received coaching on the MD dietary guidelines and met with nutritional specialists, and a 14-point MD adherence questionnaire was used to monitor adherence levels. The prostate-specific antigen, C-reactive antigen, and DNA damage were evaluated at baseline and three months after following the MD. Compared to the baseline, there was a significant reduction in the DNA damage of cells in patients who had a strong adherence to the MD (p<0.013). Meanwhile, in contrast, a high intake of red meats (p<0.003) and dairy products (p<0.008) increased the cellular DNA damage significantly. It was concluded, using the Spearman bivariate correlation, which analyzed the DNA damage with the consumption of components of the MD on the adherence questionnaire, that the MD was overall inversely associated with DNA damage [15]. 

Castello et al. conducted a multi-case control study in seven providences in Spain between 2008 and 2013, in which 754 cases of prostate cancer were identified and compared with 1,277 matching control subjects. The investigators aimed to determine the impact of MD on prostate cancer risk. The Gleason score was used, which indicates prostate cancer risk, identifying tumor aggressiveness and tumor extension. A dietary evaluation using a 154-item semiquantitative food frequency questionnaire was used to assess the MD. Consuming a rich MD consisting of fruits, vegetables, fish, legumes, and olive oil was significantly associated with an inverse relationship with aggressive prostate tumor, as indicated by a lower Gleason score (p<0.023) and tumor extension (p<0.024). The researchers concluded that the MD resulted in less aggressive prostate cancer development and determined that aspects of the MD could act as a preventative factor for disease progression in prostate cancer patients [16].

Gastric Cancer

Stojanovic et al. completed a case-control study between 2003 and 2015, following 223 patients with gastric cancer and 223 controls. Evaluation of intake was assessed through a food frequency questionnaire evaluating dietary consumption in the last year. The adherence to the MD was assessed via the MEDI-LITE score, a nine-component scoring system including categories of fruit, vegetables, legumes, cereals, fish, meat, dairy, alcohol, and olive oil. It was found that the higher the adherence to the MD, the greater the reduction in risk of developing gastric cancer. An increase of 1 unit on the MEDI-LITE score was found to result in an additional 30% risk reduction. The key dietary aspects of vegetables, legumes, and fish with a low consumption of alcohol and meat significantly reduced the risk of gastric cancer. The researchers concluded that the MD as a whole and its components could substantially impact the reduction of gastric cancer prevalence [17]. 

Alvarez-Alvarez et al. conducted a multi-case control study including 354 gastric cancer cases with 3,040 controls to evaluate the effect of the MD on gastric cancer. Five different scoring systems were used: MedDietScore, relative MD, alternative MD, Dietary Score, and the MEDI-LITE assessment. Consumption of a high adherence MD resulted in a statistically significant reduction in gastric cancer risk across all five score systems (p<0.001) among the gastric cancer subtypes of cardia, non-cardia, and adenocarcinoma. Meanwhile, no reduction was identified in diffuse gastric cancer risk. The MedDietScore and Dietary score assessments estimated a 48 percent to 68 percent odd reduction in the chances of developing gastric cancer by consuming a well-balanced and strict MD. The authors concluded that the MD could help reduce gastric cancer risk overall; however, they emphasized the need for a standardized scoring system [18]. 

Amiry et al. conducted a case-control hospital-based study in Afghanistan, which included 90 patients with gastric cancer and 180 controls. The Willett-format food frequency questionnaire, adapted for the Afghan diet, was used to evaluate the intake of each participant. Adherence to the MD was determined by dietary factors in the nine categories of fruits, vegetables, fish, legumes, nuts, whole grains, monounsaturated fatty acids, saturated fatty acids, meats, and dairy products. The investigators found a 52 percent risk reduction in gastric cancer with high adherence to the MD (p<0.05). Individuals with a high adherence had diets rich in fruit, vegetables, fish, legumes, nuts, total energy, total fat, dietary fiber, folate, and magnesium, and lower intakes of whole grains, carbohydrates, and niacin. The researchers concluded that the MD may have the additional benefits of reducing gastric cancer [19]. 

Tayyem et al. conducted a case-control study of 172 patients with gastric cancer and 314 controls to assess the impact of four dietary patterns on the malignancy: MD, prudent, unhealthy, and high-fruit diets. The MD was classified as being high in vegetables and fruits. A prudent diet includes fruit, vegetables, soup, whole bread, cereal, milk, and low or fat-free white cheeses. The unhealthy diet was focused on artificial fruit juices, cooked vegetables, cabbage salad, fried potatoes, pasta, burgers, and processed meats. The high-fruit diet was focused on only the consumption of fruit. A food-frequency questionnaire for Arabic dietary habits was used to evaluate patients' diets. It was found that consumption of the MD in the third and fourth quartile was significantly associated with a reduction in the odds ratio of gastric cancer compared to the unhealthy and prudent diet, which elevated gastric cancer risk, and the high-fruit diet which had an insignificant impact. The authors concluded that the MD plays a larger role in reducing gastric cancer than other dietary habits [20].

Colorectal Cancer

Grosso et al. conducted a case-control study evaluating 338 patients diagnosed with colorectal cancer and matched them with 676 control subjects without any cancers between 2000 and 2012. Information regarding the subject’s lifestyle and socioeconomic status were assessed. The MedDietScore was used to measure the adherence and quality of the MD. Researchers determined that commitment to the MD resulted in significant reductions in the risk of developing colorectal cancer as well the higher adherence to the MD resulted in lower severity health-related implications associated with colorectal cancer. The findings indicate the positive impact that the MD could have on not only the odds of developing colorectal cancer but also additional benefits in curbing the severity of colorectal cancer in patients presently diagnosed [21]. 

Bladder Cancer

Bravi et al. examined 690 cases of bladder cancer patients with 665 controls without any form of cancer to examine the association between MD and bladder cancer. The MD was assessed using the MedDietScore, which ranged from zero to nine, least to most adherence. Units on the MedDietScore were rewarded to patients whose intake was above average. It was determined that a high commitment to the MD in the six to nine score range would reduce 12.6 percent of bladder cancer cases (p<0.02). Higher adherence to the MD reduced the risk of bladder cancer development by 35 percent as compared to the low adherence group. Overall, Bravi et al. concluded the beneficial impacts that the MD might have on the reduction in the occurrence and risk of bladder cancer [22]. 

Lung Cancer

Gnagnarella et al. evaluated 4,336 participants in a lung cancer screening program who were current smokers and those who recently quit, assessing the MD's impact on the development of lung cancer. During the evaluation period of 5.7 years, 178 participants developed lung cancer. A self-administered food frequency questionnaire was used to calculate the Alternative MD score, ranging from zero to nine, to determine adherence to the MD. Higher adherence to the MD was significantly associated with a reduced lung cancer risk (p<0.045). Specifically, there were significant decreases in lung cancer risk with high consumption of vegetables (p<0.03), fruits (p<0.03), olive oil (p<0.01), and fish (p<0.04) and a significant increase in cancer risk with high consumption of red meat (p<0.003). The researchers concluded that MD could reduce the chances of lung cancer development in current smokers and those who have recently quit [23].

Thyroid Cancer

Barrea et al. conducted a first-of-its-kind study analyzing the relationship between MD and nodular thyroid disease and thyroid cancer. The study population consisted of 794 subjects with thyroid nodules. The classification of the nodules was completed with fine needle aspiration and divided into five categories of increasing malignancy potentiation: TIR2, TIR3a, TIR3b, TIR4, and TIR5. The Prevention with MD scoring system, a 14-item questionnaire, was used to evaluate adherence to an MD. In the study, only 15.7 percent of patients showed a high adherence as compared with 49.4 percent having an average adherence to the MD. Low adherence to the MD was classified as diets low in olive oil, vegetables, and fish, resulting in an increased presentation of thyroid nodules (p<0.001). In addition, the researchers found that patients with TIR 5 thyroid nodules, the highest risk of malignancy category, had the lowest adherence dietary pattern (p<0.001). The investigators concluded that MD adherence could be associated with limiting thyroid nodular disease and malignancy [24]. 

KD impact on cancer progression and risk

Breast Cancer

Khodabakhshi et al. evaluated 40 patients with breast cancer undergoing chemotherapy and 40 controls in a randomized clinical trial to analyze the impact of a medium-chain triglyceride KD as a supportive therapy given for 90 days. It was determined that the KD group significantly decreased tumor size by 27 mm compared to six mm and in cancer staging, referring to the change in size and location of the tumor (p<0.01). In addition, crucial inflammatory markers of TNF-alpha (p<0.001), insulin (p<0.002), and IGF-1 (p=0.02) were found to have decreased significantly while IL-10 increased (p<0.001). These findings are valuable as the downregulation of TNF-alpha, insulin, and IGF-1 limits the angiogenesis of the tumor, while the upregulation of IL-10 amplifies the anti-tumorigenic effects. The authors concluded that KD could be a beneficial therapy for patients on chemotherapy as the diet reduced inflammatory markers, tumor markers, growth factors, tumor size, and tumor stage [25]. 

Khodabakhshi et al. enrolled 60 patients with breast cancer and assigned half to the chemotherapy-only group and the ketogenic diet-chemotherapy group for 3 months. The medium-chain triglyceride KD was given for 90 days and monitored with the USDA Standard Reference Database. The overall survival rate in the KD-chemotherapy group was significantly increased compared to the chemotherapy-only group (p<0.04). It was found that blood ketones increased, and fasting blood sugar was reduced, which are both important mediators for limiting tumor growth with the introduction of the diet (p<0.001). Additionally, it positively reduced the lipid profile and renal and liver markers in patients. The authors concluded the potential effectiveness of the KD as a neoadjuvant treatment for patients with breast cancer [26]. 

Gastric Cancer

Toorang et al. conducted a case-control study at the Iran Cancer Institute between 2010 and 2012, evaluating 178 patients with gastric cancer and 276 controls in an effort to evaluate the impact of the KD on gastric cancer. A 146-item diet history questionnaire was used to assess the quality of KD. Patients with high adherence to the KD, as expected, had a significantly higher intake of fats (animal and plant) and protein and a low amount of carbohydrates (P<0.001). However, the authors found that the KD did not impact the risk of developing gastric cancer (p<0.26). It was concluded that KD does not play a beneficial role in reducing the odds of gastric cancer [27]. 

Colon Cancer

Furukawa et al. conducted a longitudinal study of 10 patients diagnosed with stage IV colon cancer to evaluate the alteration in the effectiveness of chemotherapy treatment with the addition of the KD as an adjunctive therapy. Patients were separated into three groups: The ketogenic-chemotherapy responsive group, the ketogenic-chemotherapy non-responsive group, and the chemotherapy-only group. Chemotherapy was administered in a 1.4 to 1 ratio with the medium-chain triglyceride KD. The investigators found that patients in the ketogenic-chemotherapy group had a 60 percent response rate to therapy and a 70 percent disease control rate. No overall improvement in survival rates was found; however, the survival periods of those in the chemotherapy-ketogenic responsive therapy group were longer, at 50 months, compared to 23 and 32.5 months in the ketogenic-chemotherapy non-responsive group and the chemotherapy-only group, respectively. It was concluded that adding the KD to chemotherapy may be a beneficial supportive therapy to offer patients to improve survival and the effectiveness of pharmacological treatments. Meanwhile, due to the limited number of patients in the study, future studies are needed [28]. 

Plant-based diet impact on cancer progression and risk

Breast Cancer

Sasanfar et al. examined the relationship between consuming a plant-based diet with breast cancer risk in 412 participants diagnosed with breast cancer and 456 controls. Dietary data was collected using a validated 168-item food frequency questionnaire. Diets were grouped into the classifications of a healthy plant-based diet, an unhealthy plant-based diet, and an overall plant-based diet. They found an association between the reduction in breast cancer risk with the consumption of a healthy plant-based diet (p<0.002); specifically, the participants who consumed the highest quartile of the healthy plant-based diet were associated with the lowest risk of breast cancer. These findings were maintained in both pre-menopausal and post-menopausal women. The investigators concluded that consuming a plant-based diet, especially a healthy plant-based diet, could significantly reduce the odds of breast cancer [29].

Rigi et al. evaluated the impact of a plant-based diet on 350 women with newly diagnosed breast cancer matched to 700 control subjects between 2013 and 2015. The quality of the plant-based diet was categorized into three groups: Overall consumption of a plant-based diet, a healthy plant-based diet, and an unhealthy plant-based diet. The Willett-format semi-quantitative food frequency questionnaire was used to evaluate the diet quality. The researchers found a 77 percent reduction in the risk of breast cancer in women in the highest quartile who adhered overall to a plant-based diet versus women in the lowest quartile (OR 0.23; 95% CI 0.15-0.33). Specifically, women with the highest adherence to a healthy plant-based diet experienced a 36 percent reduction in odds of developing breast cancer (OR 0.64; 95% CI 0.43-0.94). Those in the highest category consuming an unhealthy plant-based diet had a 2.12 times greater chance of developing breast cancer (OR 2.12; 95% CI 1.46-3.08). In addition, plant-based diets were found to have an inverse relationship with the development of breast cancer in both pre- and post-menopausal women. The researchers determined that plant-based diets, with a specific emphasis on healthy plant-based diets, could reduce breast cancer risk [30]. 

Colorectal Cancer

Ratjen et al. introduced a plant-based diet to 1,404 colorectal cancer survivors to analyze its impact on survival and reoccurrence rate. To determine the quality of the plant-based diet, the researchers scored healthy plant foods as whole grains, vegetables, fruits, legumes, nuts, oils, tea/coffee, and less healthy plant foods such as refined grains, fruit juices, sugar-sweetened beverages, potatoes, sweets/desserts. The plant-based diet was scored via a 112-item food frequency questionnaire over 12 months. Overall, the investigators found that the consumption of a plant-based diet displayed a significant reduction in the mortality rate of colorectal cancer survivors seven years into remission. In addition, the researchers found that a 10-point increase in the scoring of a plant-based diet results in a 28 percent reduction in mortality rate (p<0.04). Meanwhile, on the contrary, a 10-point decrease in the scoring of the quality of the plant-based diet was found to cause a higher risk of mortality among all ages and sexes (p<0.04). An additional, yet statistically insignificant finding, was that a higher-quality plant-based diet resulted in lower mortality rates, and a lower-quality planted-based diet led to higher mortality rates. Overall, the authors concluded that introducing a plant-based diet into the diet of colorectal cancer survivors could improve survival rates and overall health [31]. 

Hepatocellular Carcinoma

Liu et al. conducted a longitudinal study of 88,770 women and 48,197 men, with follow-ups every four years from 1980 to 2012, using a food frequency questionnaire to monitor the quality of plant-based diet consumed and the development of hepatocellular carcinoma. During the monitoring period, 156 participants developed hepatocellular carcinomas. Plant-based diets were seen to have an inverse relationship with hepatocellular carcinoma risk (p<0.03). Substituting carbohydrates and refined grains to plant fats within the plant-based diet guidelines further reduced hepatocellular cancer risk (p<0.001). Participants without type 2 diabetes mellitus who maintained quality plant-based diets were seen to have a lower incidence of hepatocellular carcinoma (p<0.049). The authors concluded that consumption of a plant-based diet could lead to further reductions in risk factors compared to animal-based diets. In addition, the researchers noted that substitutions within the plant-based diet could further its impact on cancer burden [32]. 

Summary

Sustaining high-quality nutrition during oncological treatments is not only a foundation to provide adequate energy to body systems to maintain sustenance but also plays a crucial role in cancer risk prevention and management of progression [4]. The transition to health-conscious diets of the Mediterranean, ketogenic, and plant-based diets has been a major influence in improving the outcomes of patients. Reviewing the literature, almost all articles concluded that the transition to one of these diets was beneficial in preventing cancer incidence or reducing progression (transition to metastases), tumor staging, tumor markers, and inflammatory markers [10-26,28-32]. Specifically, when paired with traditional chemotherapy, Khodabakhshi et al. [25,26] and Furukawa et al. [28] found significant positive implications of the addition of the ketogenic diet paired with chemotherapy rather than chemotherapy alone. Patients consuming the KD with chemotherapy were found to have reduced tumor sizes by an additional 21mm, lower cancer staging, increased anti-tumorigenic factors of IL-10, reduced factors that promote angiogenesis such as TNF-alpha, insulin, and IGF-1, and a higher survival rate [25,26,28]. Out of the 23 studies evaluated, only one study, Toorang et al. [27], did not conclude the potential effectiveness of including a dietary intervention in the potential treatment plan for oncological patients. Evaluation of the KD impact on patients with gastric cancer by Toorang et al. did not conclude significant improvements [27]. However, at the time of completion of the study and currently, it is the only article that has analyzed the KD with gastric cancer. Further research is required to provide additional evidence to support the claim.

Based on the amount of evidence presented in this review, evaluating the impact of the MD, KD, and plant-based diet on cancer burden, the MD should be the most recommended diet as a result of its extensive research and composition of dietary nutrients [6,10-24,33-35]. The MD was found to have the most research conducted on its impact on cancer prevention with fifteen articles, and it has been well-evaluated over widespread types of cancers, including gastric, breast, thyroid, colorectal, endometrial, lung, bladder, and prostate cancer [10-24] compared to four articles evaluating the KD only within gastric, colon and breast cancer [25-28] and four articles evaluating the plant-based diet amongst breast, hepatocellular, and colorectal cancer [29-32]. Evaluating each diet’s main components is additionally crucial to better understand the relationship behind the MD’s effectiveness as an adjunctive approach with oncology treatments compared to the KD and plant-based diets. The MD consists of omega-3 fatty acids, green leafy vegetables, fruits, whole grains, legumes, nuts, fish, and poultry, with a limited intake of dairy and processed red meats [6]. These substances are high in anti-proliferative, anti-inflammatory, anti-angiogenic, anti-metastatic, anti-oxidant, and pro-apoptotic factors, which have been shown to be potential leading factors behind the risk of cancer development in both in vivo and in vitro studies [33]. Specifically, fruits and vegetables contain high anti-oxidant properties of carotenoids, vitamins C and E, folates, and flavonoids [34,35]. Fish is the main protein consumed in the MD, containing a significant amount of omega-3 fatty acids, which are crucial in reducing inflammation, angiogenesis, metastasis, and cell proliferation. With the main focus on fish, the low consumption of red meat products minimizes the intake of nitrates and polycyclic aromatic hydrocarbons, which can damage cells’ DNA, leading to the potential of cancerous mutations [34,35]. The intake of whole grains supports the bulking of stool to increase transit time, reducing the potential interaction of carcinogens with normal cells and limiting the absorption of endotoxin released by bacteria which can lead to the damage of cells. In addition, fiber breaks down into short-chain fatty acids, an energy source that is unable to be used by cancer cells leading to slower growth and development [34,36]. In comparison, the ketogenic diet, a low carbohydrate and high low-density lipoprotein and cholesterol diet, has been found to have both anti-cancer and cancer-promoting factors [37-41]. The low-carbohydrate consumption results in a state of ketosis causing an absence of the body’s main energy source of glucose and the rise of ketone bodies which have been found to reduce the size of tumors, diminish tumor growth and increase the survival rates in animal studies and in vitro but this finding has not been adequately analyzed in humans [37,38]. On the other hand, the potential anti-cancer effects of the KD are combated with the high lipid environment that has been found to be cancer stimulating as cancer cells have been found to require cholesterol for proliferation and growth [39-41]. Studies have shown that reducing cholesterol biosynthesis and uptake by cancer cells with the introduction of statins leads to a greater efficacy of chemotherapeutic drugs by reducing the survival and proliferation of cancer cells [39,42,43]. Specifically, gastric cancer patients have been found to have low high-density lipoproteins, high LDL, and high cholesterol levels, which could provide an explanation behind Toorang et al. [27] finding negligible effects of the KD in patients with gastric cancer due to the high lipid intake [44]. With regard to plant-based diets, following healthy plant-based diets has been found to reduce the risk of cancer occurrence and improve the prognosis of patients; however, unhealthy plant-based diets have been found to have higher mortality rates [31,45,46]. There is a lack of homogeneity between the classifications of healthy and unhealthy plant-based diets; in addition to a lack of research investigating the transition to a plant-based diet with oncological treatments, and a large heterogeneity within study populations and research methods indicates the need for additional studies [45,47,48]. Due to the number and strength of studies that positively correlate the MD’s components to decreased cancer incidence and progression [10-24] in comparison to the KD and plant-based diets, the MD appears to be the most evidence-supported dietary option for those at an increased odds of cancer development or currently diagnosed with cancer. The effects of the ketogenic and plant-based diets should be researched further.

In addition to expanding the field of dietary treatment options, the benefits of all three diets should be evaluated in patients undergoing not only chemotherapy, the current main area of current research, but also within the rapidly growing field of immunotherapy. Immunotherapy focuses on assisting the body’s immune system in identifying, locating, and attacking cancer cells by creating durable populations of tumor-specific T-cells that can specifically target, lyse, and eradicate cancer cells instead of chemotherapy which conducts widespread immunological cell apoptosis [49,50]. Immunotherapy has been approved for numerous types of cancers, including breast cancer, lymphoma, colorectal cancer, leukemia, melanomas, Merkle cell carcinoma, renal cell carcinoma, glioblastoma, cervical cancer, epithelial ovarian cancer, fallopian tube cancer, small-cell lung cancer, non-small cell lung cancer, gastric cancer, soft tissue cancer, and gastroesophageal junction adenocarcinoma to list a few [49]. The interactions between diet and immunotherapy are yet to be widely investigated. Understanding the impact of these diets with this growing treatment method may further provide clinicians with guidance regarding dietary recommendations for additional patients.

The limitations associated with our findings are the inconsistencies of the food items, which were included in each diet within each study as well as the portion sizes, and the variation in the points assigned to each food item indicating a lack of standardization in scoring systems for all three diets. In addition, a large number of studies used food surveys that relied on participant responses. This form of obtaining information can lend itself to response bias by participants who alter their true dietary intake. Furthermore, in our analysis of ketogenic and plant-based diets, there were few studies available to analyze, which limits the ability of an in-depth evaluation; however, the reviewed articles had consistent findings. Finally, the inaccessibility of articles due to the absence of full text may have led to the omission of additional evidence currently in the literature. 

## Conclusions

Dietary patterns play an important role in cancer prevention and the effectiveness of oncological treatments. The aim of this study was to evaluate and summarize the known understanding of the current common modern diet trends on cancer burden to serve as a tool for clinicians to better inform their patients regarding recommended dietary patterns. Improvements in cancer risk and progression were noted with the consumption of Mediterranean, ketogenic, and plant-based diets. All evaluated articles, except one that assessed the connection between the ketogenic diet and gastric cancer, indicated the beneficial role each diet may have on the prognosis of various cancers. The MD thus far has been the most extensively researched in the literature amongst multiple cancer types and has shown the strongest relationship in reducing the odds of cancer development, advancement, and tumor burden. Further longitudinal research is required to evaluate the feasibility of long-term adherence to these dietary habits and their impact on the survival rates of terminally staged cancers.

Our recommendations to expand future clinical practice and patient care are to focus on the importance of dietary modifications, as key lifestyle adaptions, in treating patients to reduce cancer risk and progression. Current evidence shows that transitioning from a traditional Western-based diet to MD, KD, and plant-based diets resulted in patients experiencing decreased cancer development or increased survival rates with diminishing tumor size and metastases. For this to occur on a larger scale, it is crucial to establish standardized classifications of exact food items and portion sizes that satisfy the requirement of each diet to ensure patients have the most accurate information and clear guidelines to follow to support adherence better. Working towards standardizing recommendations will lead to identical physician recommendations and limit confusion for patients to make dietary adjustments more accessible and feasible therapy options. Furthermore, dieticians play a crucial role on the healthcare team, assisting patients in selecting meals that follow the prescribed dietary recommendations while also making them enjoyable for the patient. Physicians should work closely with dieticians to support patients in transitioning to and maintaining long-term adherence to adjunctive diets alongside oncological treatments.
